# Therapeutic miR-506-3p Replacement in Pancreatic Carcinoma Leads to Multiple Effects including Autophagy, Apoptosis, Senescence, and Mitochondrial Alterations In Vitro and In Vivo

**DOI:** 10.3390/biomedicines10071692

**Published:** 2022-07-13

**Authors:** Hannes Borchardt, Alexander Kogel, Hermann Kalwa, Ulrike Weirauch, Achim Aigner

**Affiliations:** 1 Rudolf-Boehm-Institute for Pharmacology and Toxicology, Clinical Pharmacology, Faculty of Medicine, University of Leipzig, 04107 Leipzig, Germany; hannes.borchardt@medizin.uni-leipzig.de (H.B.); ulrike.weirauch@posteo.de (U.W.); 2 Department of Cardiology, University Hospital Leipzig, 04103 Leipzig, Germany; alexander.kogel@medizin.uni-leipzig.de; 3 Rudolf-Boehm-Institute for Pharmacology and Toxicology, Faculty of Medicine, University of Leipzig, 04107 Leipzig, Germany; hermann.kalwa@medizin.uni-leipzig.de

**Keywords:** miR-506, pancreatic cancer, miRNA replacement therapy, miRNA

## Abstract

Pancreatic ductal adenocarcinoma (PDAC) is a leading cause of cancer mortality. Considering its very poor prognosis, novel treatment options are urgently needed. MicroRNAs (miRNAs) are involved in the regulation of various physiological and pathological processes. In tumors, aberrant downregulation of given miRNAs may result in pathological overexpression of oncogenes, rendering miRNA replacement as a promising therapeutic strategy. In different tumor entities, miRNA-506-3p (miR506-3p) has been ambivalently described as tumor suppressing or oncogenic. In PDAC, miR-506 is mainly considered as a tumor-suppressing miRNA. In this study, we extensively analyze the cellular and molecular effects of miRNA-506-3p replacement in different PDAC cell lines. Beyond profound antiproliferation and induction of cell death and autophagy, we describe new cellular miR506-3p effects, i.e., induction of senescence and reactive oxygen species (ROS), as well as alterations in mitochondrial potential and structure, and identify multiple underlying molecular effects. In a preclinical therapy study, PDAC xenograft-bearing mice were treated with nanoparticle-formulated miRNA-506 mimics. Profound tumor inhibition upon systemic miRNA-506 administration was associated with multiple cellular and molecular effects. This demonstrates miRNA replacement as a potential therapeutic option for PDAC patients. Due to its broad mechanisms of action on multiple relevant target genes, miR506-3p is identified as a particularly powerful tumor-inhibitory miRNA.

## 1. Introduction

Pancreatic cancer is one of the leading causes of cancer mortality, with still very poor prognosis for patients and a 5-year survival rate of only ~10% [1,2]. Pancreatic ductal adenocarcinoma (PDAC) accounts for about 90% of all cases. Since chemotherapy and surgery have not led to major improvements for patients, new targets and novel treatment options are urgently needed. Small noncoding RNAs (microRNAs, miRNAs) are substantially involved in the regulation of physiological and pathological processes. In cancer, several miRNAs have been found over- or underexpressed, which may be of functional relevance, since miRNAs, once introduced into the RNA-induced silencing complex (RISC), can sequence-specifically lead to either cleavage of their complementary mRNAs or inhibition of mRNA translation. In tumors, aberrant downregulation of a given miRNA may result in pathological overexpression of oncogenes, rendering miRNA replacement as a promising strategy in cancer therapy [3]. The identification of suitable miRNAs and the delineation of their (patho-) physiological roles, however, is a critical step and will strongly influence therapeutic success in terms of outcome for patients.

The miRNA-506-3p (miR506-3p) has been ambivalently described in different tumor entities as both a tumor suppressor miRNA or an oncogenic miRNA [4]. As a member of the miR-506-514 cluster, it was found overexpressed in melanoma, and its inhibition contributed to reduced cell growth, apoptosis induction, and decreased colony formation of melanoma cells [5]. In contrast, other studies in gastric, breast, ovarian, colorectal, and liver cancer showed miR506-3p to inhibit cell migration and angiogenesis, promote cell senescence, and reduce the metastatic potential of tumor cells [6,7,8,9,10]. Furthermore, the inhibition of epithelial-to-mesenchymal transition (EMT) by miR506-3p led to reduced tumor progression in ovarian and gastric cancer [11,12].

In pancreatic cancer, miR-506 has been mainly described as a tumor-suppressing miRNA. Du et al. demonstrated miRNA-506-3p downregulation in ~71% of all analyzed pancreatic cancer samples when compared to normal adjacent tissue. In parallel, PIM-3, a member of the proto-oncogene PIM family, was found upregulated in pancreatic cancer tissue and negatively correlated with miR-506 levels [13]. Reduced miR-506 expression was associated with pancreatic progression, while upregulated exerted inhibitory effects [14,15,16,17,18]. In this context, STAT3 [17] and sphingosine kinase 1 (SPHK1 [14]) were identified as direct targets, and miR-506 levels were described to be affected by the oncogenic lncRNA NEAT1 [15] or by rosmarinic acid [16]. In contrast, Cheng et al. analyzed by in situ hybridization (ISH) the expression of miR-506 in PDAC patients’ material vs. healthy donors and detected miR-506 upregulation in PDAC as compared to normal pancreatic ductal cells. However, poorly differentiated PDACs showed lower miR-506 levels than their moderately differentiated counterparts, and a negative association between miR-506 expression and pathologic T category was found. This led to the suggestion of miR-506 acting as an oncogene in tumorigenesis and a tumor suppressor in further tumor progression, by downregulation of the tumor suppressor gene TP53 [19].

With the aim of a possible therapeutic exploration of miR506-3p, we extensively analyze in this study the effect of miR506-3p replacement in different pancreatic cell lines in vitro and in a preclinical therapy study in vivo.

## 2. Materials and Methods

### 2.1. Cell Lines and Culture Conditions

RPMI 1640 Medium (Sigma, Taufkirchen, Germany) supplemented with 10% heat-inactivated fetal bovine serum (FBS Superior, Biochrom, Berlin, Germany) was employed to culture (37 °C, 5% CO_2_) all of the used cell lines (AsPC1, Colo357, Panc89, Panc1, PaTu-8988t). Cell lines were purchased from the American Type Culture Collection (ATCC, Manassas, VA, USA).

### 2.2. miRNA Transfection

The *C. elegans*-specific negative control miRNA NC1 (5′-UCACAACCUCCUAGAAAGAGUAGA-3′) and the human miRNA hsa-miR-506-3p mimic (5′-UAAGGCACCCUU-CUGAGUAGA-3′) were obtained from Merck (Darmstadt, Germany). The cells were seeded 24 h prior to transfection. INTERFERin transfection reagent (Polyplus Transfection, Illkirch, France) was used for transfection of the miRNAs at final concentrations of 5 nM (PaTu-8988t) or 10 nM (Colo357; Panc89).

### 2.3. Measurement of Cell Proliferation, Live/Dead Cell Staining, and Hoechst33342 Staining

The cells were transfected in 96-well plates, and proliferation was determined using CCK-8 (Dojindo EU, Munich, Germany) according to the manufacturer’s protocol. For this, CCK-8 was diluted 1:10 in serum-free medium and pipetted onto the cells prior to incubation at 37 °C and 5% CO_2_ for 1 h and measurement of viable cell numbers in a Fluostar Optima reader at 450 nm. Live/dead cell staining was performed for 30 min using propidium iodide (dead cells) and calcium-AM (viable cells), both at a concentration of 1 µg/mL prior to measurement in a Celigo Imaging Cytometer (Nexcelom Bioscience, Lawrence, MA, USA). Afterwards, Hoechst33342 (1 µg/mL) was used for bright-field staining of nuclei in order to determine the average growth area in µm².

### 2.4. RNA Preparation, Reverse Transcription, and qPCR

TRIzol^®^ (Invitrogen, Darmstadt, Germany) was used for RNA isolation, following the vendor’s protocol. Isolated RNA was subsequently transcribed in cDNA using 1 µg total RNA and the RevertAid RT Kit (Thermo Fisher Scientific, Waltham, MA, USA). The StepOnePlus™ Real-Time System (Thermo Scientific, Darmstadt, Germany) and the PerfeCTa^®^ SYBR^®^ Green FastMix^®^ ROX (QuantaBio, Hilden, Germany) were used for quantitative PCR. Quantitative real-time PCR was performed under the following conditions: 95 °C for 10 s, followed by 50 cycles comprising incubation at 95 °C for 10 s, 55 °C for 10 s, and 65 °C for 10 s, with parallel amplification of beta-actin serving as housekeeper gene. MiRNA levels inside the cells were quantitated using the qScript^®^ microRNA cDNA Synthesis Kit (Quantabio, Beverly, MA, USA). SNORD44 served as the housekeeper RNA. For primer sequences, see Table S1.

### 2.5. Western Blot

Cells were cultivated in 6-well plates for 72 h (PaTu-8988t) or 120 h (Colo357) after transfection and then lysed with RIPA buffer (20 mM Tris-HCl, pH 7.5, 150 mM NaCl, 1% NP-40, 1% sodium deoxycholate), supplemented with Protease Inhibitor Cocktail Set III, EDTA-Free (Merck). For tumor tissue analyses, the weights of the tumor xenografts were measured, and 100 µg tissue was mixed with 1.5 mL RIPA buffer prior to incubation for 30 min on ice. Tissue homogenization was performed using an ULTRA Turrax (IKA, Staufen, Germany); the homogenate was transferred to a 1.5 mL tube and further incubated on ice for 10 min. Subsequently, the homogenate was centrifuged three times at 10,000× *g* for 5 min, with transfer of the supernatant into a clean reaction tube after each centrifugation step. The clear supernatant was used to measure the protein concentration (BCA Protein Assay, Thermo Fisher Scientific) and stored at −80 °C. Samples containing 25 µg protein were separated by SDS-PAGE and transferred onto a 0.2 µm nitrocellulose membrane (GE Healthcare, Freiburg, Germany) by electroblotting. The membrane was blocked with 5% milk in TBS-T (Tris-buffered saline containing 0.1% Tween 20) for 60 min and subsequently incubated with the primary antibody overnight at 4 °C (see Table S2 for details on antibodies and dilution factors). The next day, the blots were washed three times in TBS-T, incubated with the appropriate horseradish peroxidase-conjugated secondary antibody for 60 min, and washed again three times with TBS-T. Lastly, protein bands were revealed by chemiluminescence using the SuperSignal^TM^ West Femto Maximum Sensitivity Substrate (Thermo Fisher Scientific) and scanned in an ImageQuant LAS-4000 chemiluminescence detection system (GE Healthcare, Munich, Germany). Densitometric quantitation was performed using the ImageJ software, with bands normalized to beta-actin. Details on the antibodies are given in Table S2.

### 2.6. Spheroid Formation

Pretransfected Panc89 cells (24 h, 5000 cells/well) were seeded into an ultra-low-attachment Greiner CELLSTAR 96-well-plate (Sigma-Aldrich, Munich, Germany). Spheroids were documented over several days using the Celigo Imaging Cytometer and analyzed after 72 h regarding formation and growth. Subsequently, live/dead cell staining of the spheroids was performed using propidium iodide and calcein-AM as described above.

### 2.7. Determination of Apoptosis, Necrosis, and Autophagy

For flow-cytometry-based analyses to quantify necrotic and apoptotic cells, the FITC-Annexin-V assay (Invitrogen, Carlsbad, CA, USA) was used according to the manufacturer’s protocol. To further analyze the miRNA effect on apoptosis, a bioluminescence caspase-3/7 assay (Caspase-Glo-3/7 assay; Promega, Walldorf, Germany) was performed. Briefly, the cells were transfected as described above, and 50 µL Caspase-3/7-Glo reagent, diluted 1:10 in serum-free medium, was added to the cells. After incubation for 1 h at room temperature in the dark, signals were measured in a Fluostar Optima reader (BMG Labtec, Ortenberg, Germany). In parallel, viable cells were measured on the same plate by CCK-8 as described above for calculating the ratio “caspase activity/CCK-8 signal” and thus for adjusting the caspase signal to different cell densities.

The CYTO-ID^®^ Autophagy detection kit 2.0 (ENZO Biochem, Farmingdale, NY, USA) was used to determine the miRNA effect on autophagy induction according to the manufacturer’s protocol. MiRNA-transfected cells were measured by flow cytometry. The treatment of cells with 0.5 µM rapamycin in combination with 100 µM chloroquine for 24 h served as positive control.

The autophagic flux assay was performed in 6 wells. PaTu-8988t cells were transfected for 24 h with miRNA506-3p or NC1 prior to adding bafilomycin A (100 nM) for 8, 24, or 48 h as indicated, followed by cell lysis and analysis of p62 levels by Western blot.

### 2.8. Cell Cycle Analyses and Determination of ROS

Cell cycle analyses were performed after 48 h and 72 h of transfection. For blocking the ongoing cell cycle in G2/M phase, cells were treated with 25 ng/mL nocodazole for 20 h in standard cell culture medium prior to the experimental termination. Cells were trypsinized and fixed in ice-cold ethanol (70%), washed, and incubated for 1 h in 150 µL RNaseI (50 ng/mL) in FACS solution (Attune™ Focusing Fluid, Thermo Scientific, Darmstadt, Germany). Then, 350 µL PI/FACS solution (2 mg/mL) was added to the reaction tube and incubated on ice until measurement in an Attune^®^ Acoustic Focusing Cytometer (Thermo Fisher Scientific).

For the detection of reactive oxygen species (ROS) in live cells, the CellROX™ Green Flow Cytometry Assay Kit (Thermo Fisher Scientific) was used according to the manufacturer’s protocol.

### 2.9. Senescence Induction and Measurement of Mitochondrial Potential and Structure

Induction of senescence was measured at 48 h and 72 h after transfection using the CellEvent™ Senescence Green Detection Kit (Thermo Fisher Scientific). Assays were performed according to the manufacturer’s protocol and measured by flow cytometry. For mitochondrial depolarization and mass measurement, MitoView^TM^ 633 was used at a final concentration of 100 nM and measured by flow cytometry according to the manufacturer’s protocol.

### 2.10. Confocal Imaging

PaTu-8988t cells were seeded on coverslips in a 6-well plate and transfected as described above. After 72 h of transfection, the cells were stained for senescence using the CellEvent™ Senescence Green Detection Kit (Thermo Fisher Scientific) and CYTO-ID^®^ Autophagy detection kit 2.0 (ENZO Biochem, Farmingdale, NY, USA) according to the manufacturer’s protocol. For mitochondrial live-cell staining, the dye MitoView^TM^ 633 was diluted in prewarmed RPMI medium at a concentration of 100 nM MitoView^TM^ 633. Cells were incubated at 37 °C and 5% CO_2_ for 15 min. All stainings were imaged by using the LSM 510 META confocal microscope (Carl Zeiss, Oberkochen, Germany) and analyzed/visualized by using ImageJ software.

### 2.11. Xenograft Mouse Model and Nanoparticle-Based Therapy

All mouse experiments were performed strictly following the national regulations of animal welfare, with prior ethical review and approval by the local committee on animal welfare and the local authorities (Landesdirektion Sachsen, Leipzig, Germany). The mouse experiments were in accordance with the EU Directive 2010/63/EU for animal experiments and all relevant institutional and national guidelines, in particular the ARRIVE guidelines. SCID mice (NOD/SCID/IL2r gamma (null); Jackson Laboratories, Bar Harbor, ME, USA) were provided by the animal core facility of the University of Leipzig (Medizinisch-Experimentelles Zentrum (MEZ)) and kept in a humidified atmosphere at 23 °C and a 12 h cycle of light and dark. Standard rodent chow (ssniff, Soest, Germany) was used for animal feeding, and water was provided ad libitum.

The xenografts were established by subcutaneous (s.c.) injection of 5 × 10^6^ PaTu-8988t cells in 150 µL PBS into both flanks of the mice. When tumors reached a volume of approximately 50 mm³ and showed solid tumor growth, mice were randomized into treatment groups of *n* = 12 tumors per group, and treatment was started by intraperitoneal injection of nanoparticle-formulated miRNA-506-3p or NC1-miRNA, respectively. For nanoparticle preparation, 10 µg miRNA mimic or NC1 was dissolved in 75 µL 10 mM HEPES, 150 mM NaCl (pH 7.4), and incubated for 10 min. Likewise, 50 µg PEI F25-LMW [20] was diluted in the same buffer and incubated for 10 min prior to mixing with the miRNA solution and complexation for 15 min. The complexes were stored at −80 °C and thawed/kept for 30 min at room temperature prior to use. Complexes were injected 3× per week, and tumor volumes were measured at the same time. At the end of the experimental procedure, mice were sacrificed, tumors were removed, and their weight was measured. The tumors were divided into pieces and snap-frozen for RNA and protein or fixed in 4% paraformaldehyde prior to paraffin embedding.

### 2.12. Immunohistochemistry

MYC, survivin, Ki-67, and active caspase-3 expression was analyzed by immunohistochemistry using the antibodies detailed in Table S2. From paraffin-embedded tumors, 5 µm sections were prepared and baked at 56 °C overnight, with subsequent microwave treatment for 5 min (800 W). This was followed by deparaffinization (NeoClear, Roth, Karlsruhe, Germany), rehydration in a graded ethanol series, and epitope retrieval using the antibody-specific buffers given in Table S3. After 3× washing in PBST (10 mM phosphate, 150 mM NaCl, 0.1% Tween-20), endogenous peroxidases were blocked by 30 min incubation in 2% H_2_O_2_ in PBST at 4 °C. After three more washing steps in PBST and blockage with 2% BSA/10% normal serum in PBST, the primary antibody was added and incubated at 4 °C overnight. The next day, slides were washed three times and incubated with a biotinylated secondary antibody. After washing, incubation with an avidin–HRP complex (VECTASTAIN ABC kit, Vector Laboratories, Burlingame, CA, USA) and three more washing steps, brown staining was developed by incubating the slides in a diaminobenzidine/H_2_O_2_ solution. The slides were then counterstained with hematoxylin and embedded prior to scanning with an Axio Scan.Z1 slide scanner (Zeiss, Oberkochen, Germany).

### 2.13. Statistical Analyses

Unless indicated otherwise, all experiments were performed at least in triplicates. Statistical differences between NC1 and miRNA506-3p were calculated by using Student’s *t*-test or the Wilcoxon–Mann–Whitney test, with significance levels as follows: * *p* < 0.05, ** *p* < 0.01 and *** *p* < 0.001.

## 3. Results

### 3.1. Decreased Cell Proliferation and Viability upon Transient miR-506 Transfection

**Figure 1:**
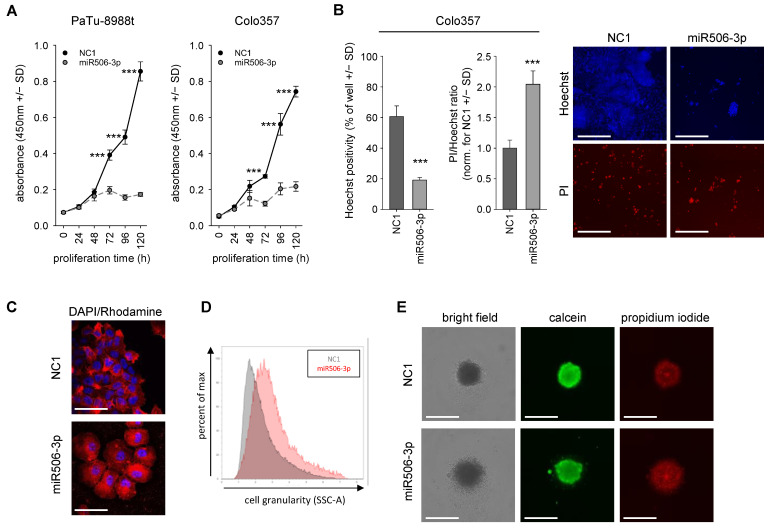
(**A**) Two-dimensional proliferation assay in PaTu-8988t and Colo357 cells after miR506-3p (gray, dashed) vs. negative control (NC1) transfection (black). (**B**) Effect of miR506-3p on cell death, visualized by propidium iodide (PI) staining for dead cells, with Hoechst counterstaining for nuclei and cell number. (**C**) Confocal imaging of miR506-3p effect on flattened cell morphology, with DAPI nuclear staining and phalloidin-rhodamine cytoskeletal staining. (**D**) Flow cytometry-based analysis of cell granularity after miR506-3p (red) vs. NC1 transfection (gray). (**D**) Altered surface shape of Panc89 spheroids, pre-transfected for 24 h with miR506-3p vs. NC1 and then cultivated for 72 h in low-adhesion plates. Spheroids are shown in bright field, as well as stained with calcein (viable cells) and PI (dead cells). Scale bars: (**B**) 300 µm; (**C**) 50 µm; (**E**) 200 µm. *** *p* < 0.001.

By RT-qPCR, miR506-3p expression levels were determined in five different pancreatic carcinoma cell lines. No major differences were observed in the cell lines AsPC1, Colo357, Panc1, and Panc89, while 3-fold higher amounts of miRNA-506-3p were observed in PaTu-8988t cells (Figure S1A). In 2D cell culture, anchorage-dependent proliferation was analyzed in Colo357 cells and in the particularly high-expressing PaTu-8998t cell line. Upon miR506-3p transfection, cell proliferation was reduced by up to ~80% compared to negative control miRNA NC1 (Figure 1A).

This profound antiproliferative effect was independent of initial miR506-3p expression levels. The cell-inhibitory potential of miR506-3p was confirmed in a second 2D growth assay with Celigo Imaging Cytometer analysis. Colo357 cell transfection with miR506-3p led again to markedly reduced proliferation compared to NC1, as shown by Hoechst33342 staining after 120 h (Figure 1B). Propidium iodide counterstaining for dead cells and the calculation of PI/Hoechst ratios also indicated a 2-fold increase in PI-positive cells in the specific miR506-3p treatment group. Notably, miR506-3p transfection of Colo357 cells did not affect cell morphology (data not shown), while in PaTu-8988t cells, a flattened morphology was observed (Figure 1C). This was associated with considerably increased cell granularity, as also confirmed by flow cytometry (Figure 1D). To mimic a more in vivo-like situation, pretransfected cells were seeded in ultra-low-adhesion plates to analyze the ability for spheroid formation in a 3D cell culture model. For this experiment, Panc89 cells were employed, which allow for spheroid formation under these conditions. While upon transfection with the negative control miRNA (NC1) spheroids with sharp edges were observed, miRNA-506-3p transfection led to indistinct edges due to the presence of loose, less tightly bound, or even scattered cells. Moreover, staining for live and dead cells after 72 h of treatment revealed an increase in PI-positive dead cells in the specific treatment group, while calcein positivity indicative of viable cells was slightly reduced (Figure 1E).

### 3.2. MiR-506 Induces Cell Death by Apoptosis, Autophagy and Senescence Induction, and Affects Mitochondrial Potential and Structure

**Figure 2:**
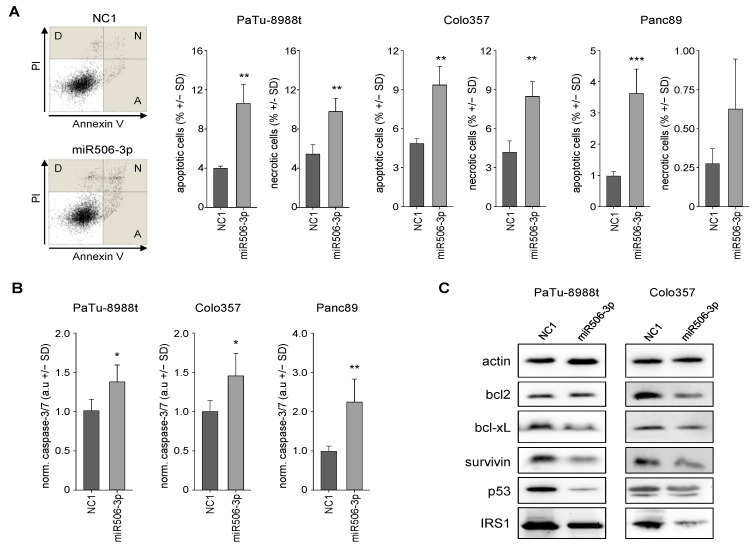
Analysis of apoptotic and necrotic fractions of PaTu-8988t, Colo357, and Panc89 cells, transfected with miR506-3p vs. NC1, by flow cytometry. (**A**) Original data from the cell line PaTu-8988t with apoptosis (A), necrosis (N), and debris (D) indicated. (**B**) Quantitation of apoptotic and necrotic cell fractions in PaTu-8988t, Colo357, and Panc89 cell samples. (**C**) Caspase-3/7 activation in PaTu-8988t (72 h), Colo357 (120 h) and Panc89 (72 h) cells, upon transfection with miR506-3p vs. NC1. (**D**) Western blot analyses of alterations in various apoptosis-related genes in PaTu-8988t (72 h) and Colo357 (120 h) cell lysates after miR506-3p vs. NC1 transfection. Expression levels of proteins related to antiapoptosis (bcl2, bcl-xL, p53, IRS-1) and survivin in PaTu-8988t (**left**) and Colo357 cells (**right**). * *p* < 0.05, ** *p* < 0.01 and *** *p* < 0.001.

Transient transfection of PaTu-8988t cells with the miR506-3p mimic led to a ~3-fold increase in apoptotic cells and a doubling of necrotic cells, as determined by flow cytometry analyses after annexin V/PI staining (Figure 2A left). Similar results were obtained in other cell lines, with somewhat more profound effects in Panc89 cells (Figure 2A right and Figure S2). This was in line with caspase-3/7 activation, which was again seen in all cell lines (Figure 2B). The direct comparison revealed again a most profound >2-fold increase in Panc89 cells, indicating that the caspase-3/7 activation is dependent on the cell line but independent of endogenous miR506-3p expression levels.

Further analyses of apoptosis-related genes revealed the downregulation of the antiapoptotic proteins bcl 2 and bcl-xL, as determined in Western blots at 72 h (PaTu-8988t cells) or 120 h after transfection (Colo357 cells; Figure 2C). Effects were again dependent on the cell line, with bcl2 effects being more profound in Colo357 cells, while bcl-xL reduction was mainly seen in PaTu-8988t cells. Moreover, the proto-oncogenic survival kinase survivin was significantly downregulated in these PDAC cell lines as well. P53, which is associated with several cellular stress responses like apoptosis, senescence, and autophagy, was downregulated in Colo357 cells and to a much greater extent in PaTu-8988t cells.

**Figure 3:**
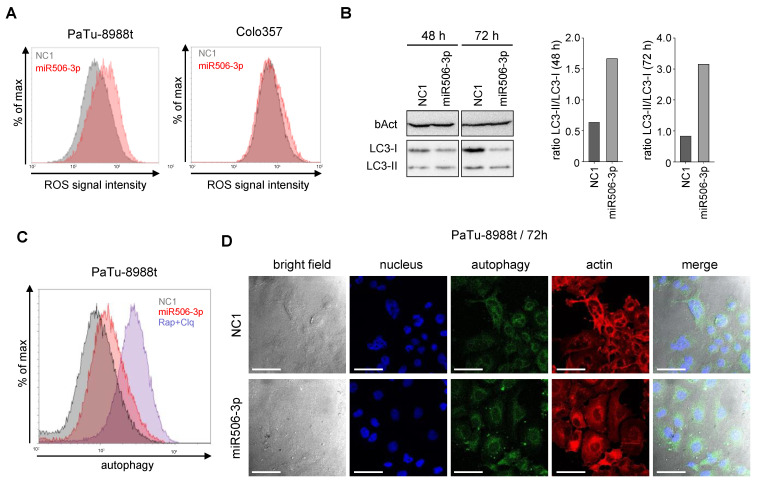
(**A**) Determination of ROS levels in PaTu-8988t (**left**) and Colo357 cells (**right**) by flow cytometry after transfection with miR506-3p (red) vs. NC1 (gray). (**B**) Time-dependent induction of autophagy upon miR506-3p, as determined by LC3-I/LC3-II Western blotting (**left**) and calculation of LC3-I/LC3-II ratios (**right**). (**C**) Induction of autophagy upon transfection of PaTu-8988t cells with miR506-3p (red) vs. NC1 (gray); blue: rapamycin + chloroquine as positive control. (**D**) Confocal microscopy of PaTu-8988t cells stained in parallel for autophagy (CYTO-ID^®^ Autophagy Detection kit 2.0; green), actin (phalloidin-rhodamine) and nuclei (DAPI; blue), upon transfection with miR506-3p (lower panel) vs. NC1 (upper panel). Scale bars: 50 µm.

Reactive oxygen species (ROS) are regulators of intracellular signaling, with the potential of inducing mitochondria-mediated apoptosis. More specifically, an increase in ROS may lead to a deregulation of intracellular redox homeostasis, and subsequent activation of cellular apoptosis pathways. Indeed, PaTu-8988t cells showed increased ROS levels at 72 h after transfection with the miRNA-506-3p mimic, while little effects were seen in Colo357 cells (Figure 3A).

PaTu-8988t cells were further analyzed on the mRNA level for ROS-related genes. Glutamic-oxaloacetic transaminase 1 (GOT1) plays an important role in energy metabolism and ROS balance [21], and its expression status is a prognostic marker in PDAC [22,23]. Notably, in our experiments, GOT1 was 3-fold upregulated in PaTu-8988t cells upon miR506-3p transfection (Figure S1B). Nuclear factor erythroid 2-related factor 2 (Nrf2), which plays an important role in resistance to oxidative stress, was found slightly increased as well. Likewise, miR506-3p transfection led to the upregulation of CK2a, which inhibits apoptosis and intracellular ROS generation, and PTGR2, which promotes cell proliferation and inhibits ROS induction. Overall, these findings demonstrated the induction of ROS and apoptosis upon miR-506-3p transfection, despite the upregulation of genes counteracting this process (Figure S1B).

Besides the apoptotic pathway, autophagy, as a nonapoptotic self-degradation process, plays an important role in cell survival and cell cycle arrest. MiR506-3p transfection revealed profound autophagy induction, as seen in Western blot analyses from PaTu-8988t cell lysates taken at 48 h or 72 h after transfection. The ratio between the autophagic vesicle-associated LC3-II and the cytosolic LC3-I is an important marker for autophagy induction and was found >2–3-fold increased in the miR506-3p-transfected cells (Figure 3B). In line with this, flow-cytometry-based autophagy measurement at 72 h after miR506-3p transfection in PaTu-8988t cells revealed a ~40% increase in autophagy (Figure 3C). Using confocal imaging, an increase in autophagic vesicles inside the cell was found after miR506-3p treatment (white arrows, Figure 3D).

For further studying autophagic flux, we employed p62/SQSTM1 as a marker. This protein is incorporated into autophagosomes and degraded in autolysosomes. Thus, elevated or decreased levels of p62 indicate reduced or enhanced autophagy, respectively [24,25]. Additionally, we used bafilomycin A as an inhibitor of autophagosome/lysosome fusion and autolysosome activity. In the absence of an inhibitor, miR506-3p led to a >50% reduction of p62 levels over NC-transfected cells already at 32 h after transfection (Figure S3A,B, left bars). This confirms the stimulating effects of miR506-3p on autophagy previously observed in the LC3-I/II assay (see Figure 3B). As expected, the addition of bafilomycin A led to a time-dependent increase in p62 levels in NC1-transfected cells, as a consequence of its inhibitory effects on autophagosome/lysosome fusion and autolysosome activity leading to decreased p62 degradation and thus p62 accumulation over time (Figure S3). Still, however, even in the presence of bafilomycin A, the counteracting, miR506-3p-mediated induction of autophagy was observed, as indicated by lower p62 levels in the miR506-3p-transfected as compared to the corresponding NC1-transfected cells. Notably, this at least partial compensation of bafilomycin A-mediated inhibition of autophagy became increasingly prominent over time, with only ~15% difference between NC1 and miR506-3p at 32 h, ~42% at 48 h, and ~70% at 72 h (Figure S3A, right and Figure S3B, rightmost bars). Thus, this experiment also demonstrates the time kinetics of miR506-3p after transfection, as well as prolonged miR506-3p-mediated autophagy induction.

**Figure 4:**
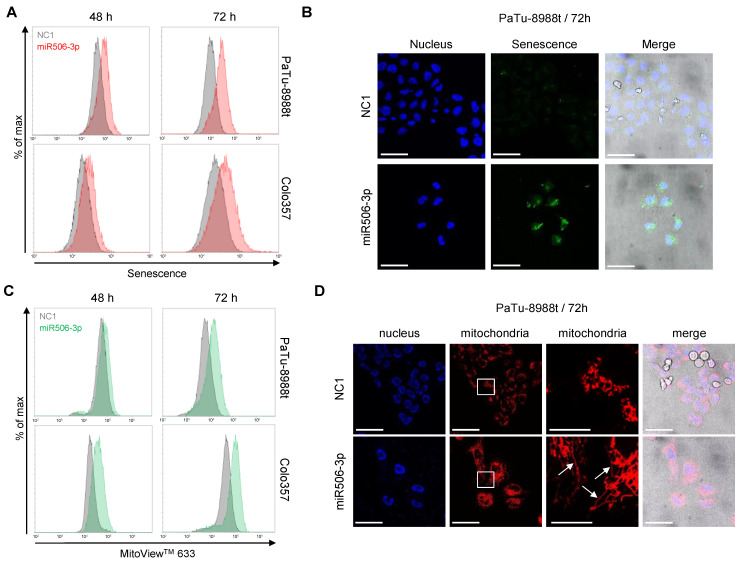
(**A**) Induction of senescence after transfection of PaTu-8988t (upper panel) or Colo357 cells (lower panel) with miR506-3p (red) vs. NC1 (gray), as determined by flow cytometry. (**B**) Confocal microscopy of PaTu-8988t cells transfected with miR506-3p (lower) vs. NC1 (upper panel) and stained with the senescence detection kit (green) and DAPI (nuclei; blue). (**C**) Flow cytometry-based determination of accumulation efficacies of the dye MitoView 633 in the mitochondrial membrane after transfection with miR-506-3p (green) vs. NC1 (gray) in PaTu-8988t (upper panel) or Colo357 cells (lower panel). (**D**) Confocal microscopy of PaTu-8988t cells transfected with miR506-3p (lower) vs. NC1 (upper panel) and stained with DAPI (**left**) and MitoView 633 (red; center panels). Boxes: areas selected for higher magnification; arrows: elongated mitochondria. Scale bars: 50 µm, higher mitochondria magnification: 15 µm.

In flow cytometry-based assays and confocal imaging, miR506-3p-transfected cells were also characterized regarding senescence and mitochondrial toxicity. Flow cytometry revealed in both PaTu-8988t and Colo357 cells a time-dependent increase in senescence at 48 h and 72 h after transfection (Figure 4A).

This finding was confirmed via confocal imaging of PaTu-8988t cells at 72 h after transfection (Figure 4B). Additionally, transfection with miRNA506-3p led to mitochondrial hyperpolarization, as determined by the accumulation of the dye MitoView 633 in the mitochondrial membrane. By flow cytometry, this was seen time-dependently in both cell lines, with an earlier onset in Colo357 as compared to PaTu-8988t cells (Figure 4C). Additionally, confocal imaging of PaTu-8988t cells stained with the same dye revealed major alterations in the morphology of the mitochondria (Figure 4D). More specifically, a shift from a fragmented to an elongated tubular structure of the mitochondria was observed (Figure 4D; see third panel for higher magnification, white arrows).

### 3.3. Induction of Cell Cycle Alterations after miRNA-506-3p Replacement

**Figure 5:**
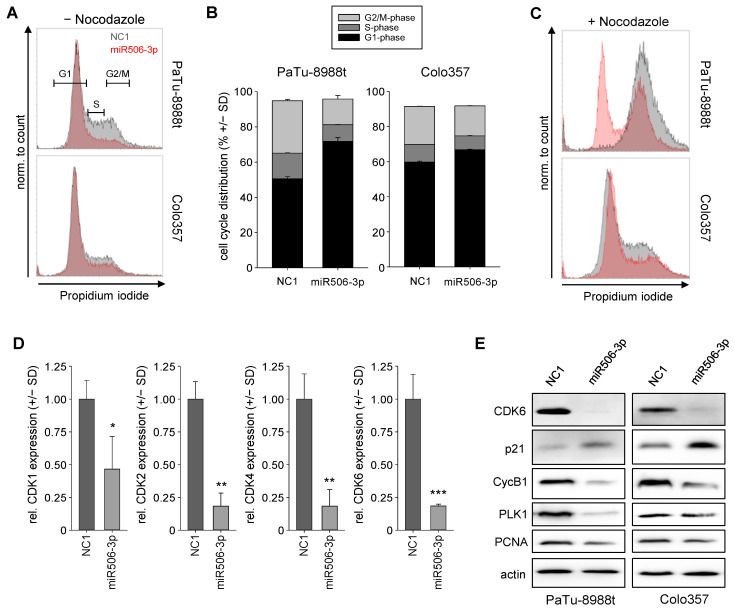
(**A**) Analysis of cell cycle distribution in PaTu-8988t (upper) cells and Colo357 cells (lower panel) after miR506-3p (red) vs. NC1 transfection (gray). **(B**) Quantitation of cell cycle distribution in PaTu-8988t (**left**) cells and Colo357 cells (**right**). (**C**) Histograms of cell cycle distribution upon transfection of PaTu-8988t (upper) cells and Colo357 cells as above + treatment with nocodazole for inducing a G2/M arrest 52 after transfection and 20 h before analysis. (**D**) Relative mRNA levels of cell-cycle-related cyclins CDK1, CDK2, CDK4, and CDK6 after miR506-3p vs. NC1 transfection. (**E**) Western blot analyses of cell-cycle-related proteins in PaTu-8988t (**left**) and Colo357 cell lysates (**right**) after miR506-3p vs. NC1 transfection. * *p* < 0.05, ** *p* < 0.01 and *** *p* < 0.001.

The transfection of miR506-3p led to profound alterations in cell cycle, as determined by flow cytometry of propidium iodide stained cells. More specifically, 72 h after transient transfection of PaTu-8988t and Colo357 cells, a marked decrease in the percentage of cells in S or in G2/M phase was observed (Figure 5A, compare gray to red histogram curves). The quantification of cell cycle distribution revealed these effects to be more profound in PaTu-8988t cells (Figure 5B). In a further experiment, nocodazole was added 20 h prior to experimental termination for inducing a G2/M blockage. This allowed for measuring the proportion of cells reaching the G2/M block in a given time period, thus indicating possible alterations in cell cycle speed upon miR506-3p transfection. Indeed, within 20 h 100% of the negative control transfected PaTu-8988t cells reached the G2/M block, while in the same time period, this was true only for a ~50% fraction of their miR506-3p-transfected counterparts, thus indicative of a considerable deceleration of cell cycle progression (Figure 5C). Again, this was particularly true for PaTu-8988t cells, which showed an overall faster cell cycle progression as compared to Colo357 cells.

In line with these miR506-3p effects on cell cycle progression, analyses of cell-cycle-dependent kinases (CDKs) showed a very profound 2–5-fold reduction of CDK1, CDK2, CDK4, and CDK6 in PaTu-8988t cells (Figure 5D), while only lesser or no alterations were observed in Colo357 cells (Figure S1C). Other cell-cycle-related proteins were further studied by Western blot analyses of lysates from NC1 vs. miR506-3p-transfected PaTu-8988t and Colo357 cells. After transfection with miR506-3p, the mediator of cell cycle progression and G1-/S-phase transition, CDK6, was almost completely depleted in both cell lines (Figure 5E, upper panel). In parallel, cyclin B1 (CycB1), which is often upregulated in cancer and leads to the transition of the cells from G2 into M phase, was markedly decreased. On the contrary, the regulator protein p21, which can inhibit the CDK4/CDK6 complex, was found markedly upregulated upon miR506-3p replacement. Another important regulator in the early phase of G2/M transition is polo-like kinase 1 (PLK-1). Here, transfection of miR506, either in PaTu-8988t or Colo357 cells, led to a marked reduction in PLK-1 expression as well. Moreover, the proliferating cell nuclear antigen (PCNA), which is active in all stages of the cell cycle, influencing DNA replication and cell cycle progression and representing a well-established marker for cell proliferation, was found reduced after miR506-3p transfection (Figure 5E). Taken together, transient transfection of miRNA-506-3p is able to slow down cell cycle progression by affecting several cell-cycle-associated proteins.

### 3.4. Nanoparticle-Formulated miRNA-506-3p Mimic Inhibits the Growth of Xenografts

**Figure 6:**
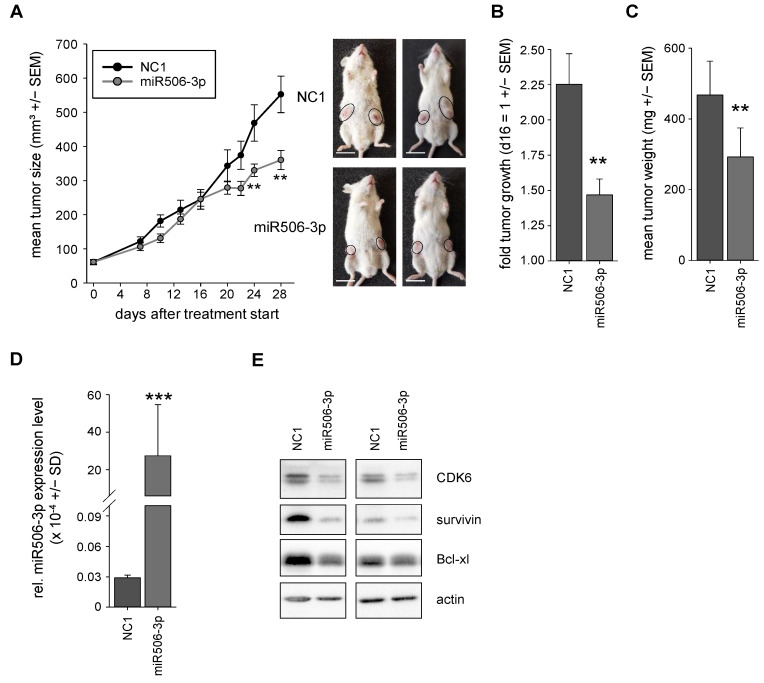
(**A**) Tumor growth curves of the in vivo therapy study in PaTu-8988t xenograft-bearing mice, treated with PEI F25-LMW nanoparticle-formulated miRNA506-3p (gray) vs. negative control miRNA (NC1, black). Right: representative pictures of tumor-bearing mice. (**B**) Tumor growth after day 16 of the specific treatment groups NC1 and miR506-3p. (**C**) Results of weight measurements of explanted tumors for cross-validation of the tumor sizes after the end of the experiment. (**D**) MiR506-3p levels in the explanted tumor xenografts upon termination of the experiment for validation of miR506-3p delivery into the tumor. (**E**) Determination of various indirect targets of miR506-3p in the lysates of the explanted tumors. ** *p* < 0.01 and *** *p* < 0.001. Scale bar: (**A**) 1.5 cm.

To test the miR506-3p in a clinically more relevant therapeutic in vivo situation, a tumor xenograft therapy study was performed. PaTu-8988t cells were subcutaneously (s.c) injected into the flanks of immunocompromised mice for inducing s.c. tumor xenografts. Upon establishment of tumors with solid growth kinetics, the mice were randomized into NC1 and specific miRNA-506-3p treatment groups. For therapeutic application, miRNAs were formulated in nanoparticles based on PEI F25-LMW [26]. These nanoparticles had already been shown previously to be efficient for miRNA delivery in vivo upon systemic administration and to be associated with high biocompatibility. More specifically, even upon repetitive treatment with PEI/miRNA nanoparticles, mice weights had been found unaltered during the whole treatment, and no other unwanted side effects (signs of discomfort, behavioral alterations), increases in liver enzymes, or induction of an immune response had been detected [20]. Concomitantly, no evident side effects were observed in this study. The nanoparticles were stored frozen at −80 °C and thawed at room temperature prior to intraperitoneal injection of the complexes containing 10 µg miRNA 3×/week. After 4 weeks, the mice of the negative control group treated with PEI-complexed nonspecific miRNA showed a ~10-fold increase in tumor volume. In contrast, upon systemic treatment of the mice with PEI/miR506-3p complexes, antitumor effects were observed, as indicated by a significant inhibition of tumor growth (Figure 6A,B). Notably, the onset of this visible tumor inhibition was observed only after 16 days of treatment. Then, however, a profound ~65% inhibition was determined until the end of the experiment at Day 28.

Upon termination of the experiment, tumors were explanted and tumor-inhibitory effects seen in the growth curves were confirmed by measurement of the tumor weights (Figure 6C). This was due to a substantial, almost 1000-fold increase in miR506-3p levels in the specific treatment group, as determined by RT-qPCR (Figure 6D). Efficient miR506-3p replacement was associated with molecular effects, which were comparable with or sometimes even exceeded the previous in vitro results. More specifically, lysates from the harvested tumors were analyzed by Western blotting and showed downregulation of CDK6, survivin, and bcl-xL on the protein level (Figure 6D).

**Figure 7:**
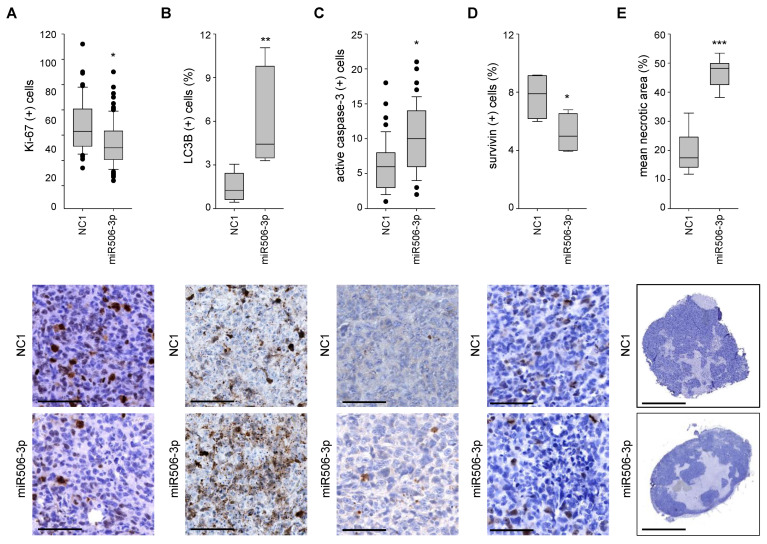
Sections of explanted tumors, immunohistochemically stained with antibodies against (**A**) Ki-67, (**B**) LC3B (**C**) active caspase-3, and (**D**) survivin. (**E**) Necrotic areas (light blue) relative to the whole tumor. Upper bar graphs: results of overall quantitation; lower panels: representative microscopic pictures. * *p* < 0.05, ** *p* < 0.01 and *** *p* < 0.001. Scale bars: (**A**–**D**) 100 µm; (**E**) 2.5 mm.

Important cellular and molecular effects were further analyzed by immunohistochemistry. While proliferation was slightly reduced in the miR506-3p treatment group, as indicated by staining for the proliferation marker protein Ki-67 (Figure 7A), the induction of apoptosis, necrosis, and autophagy was more profound and probably the main drivers of the observed antitumor effects. More specifically, using an anti-LC3B antibody with preferred detection of the autophagic vesicle-bound form LC3-II, autophagy was found >3-fold increased after the miR506-3p treatment compared to the control group (Figure 7B). Furthermore, active caspase-3 was nearly doubled, indicating apoptosis induction (Figure 7C). In line with this, reduced levels of the survival protein survivin were detected (Figure 7D). Concomitantly, histological examination of the tumor xenografts at lower magnification revealed a marked, almost 3-fold increase in necrosis in the tumors treated with a specific miR506-3p mimic (Figure 7E). This substantially larger extent of necrotic areas also indicates that the determination of tumor volumes during the study or of tumor weights at the endpoint rather underestimates antitumor effects of miR506-3p replacement. It may also explain the delayed effects on tumor sizes as downstream of the earlier onset of cellular and molecular effects leading to necrosis.

## 4. Discussion

**Figure 8:**
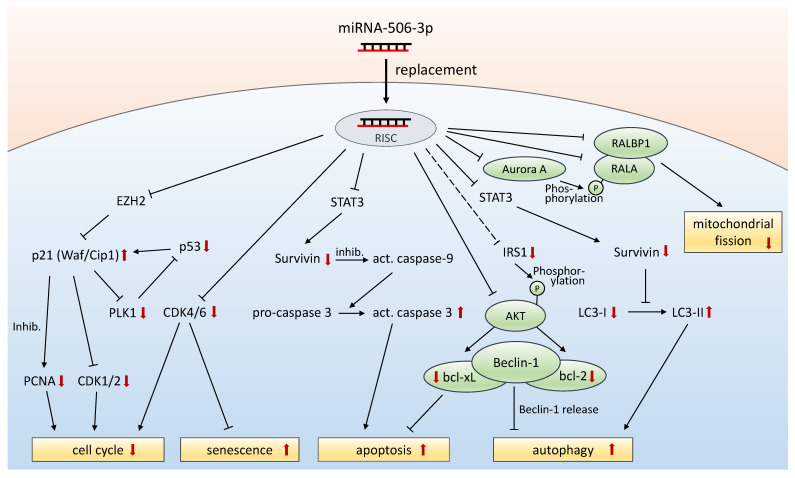
Schematic depiction of the interplay of miR506-3p effects on various direct and indirect targets. Black arrows indicate effects on expression levels or cellular processes, unless indicated otherwise. Red arrows indicate alterations in expression levels upon miR506-3p replacement, as analyzed in this study. Inhib. = inhibition.

Depending on the tumor type and context, the functional role of miR-506 has been described as ambiguous. In PDAC, most evidence indicates a tumor-inhibitory role of miR-506, suggesting, despite some inconsistency regarding miR-506 expression levels in PDAC vs. normal tissue and context-dependent opposite roles in other tumor entities, a potentially beneficial role of therapeutic miRNA replacement. While earlier studies already indicated miR-506 effects on cell cycle, cell proliferation, and apoptosis, and in part linked these findings to direct target genes, we demonstrate here that PDAC tumor-inhibition by miR506-3p is in fact mediated by several additional, important cellular and molecular mechanisms. A schematic depiction of the interplay of miR506-3p effects on various direct and indirect targets is given in Figure 8 and/or discussed below.

The direct comparison between in vitro and in vivo data, based on the same cell line, also reveals that major contributors to tumor cell inhibition in vitro may not necessarily be of similar relevance in the in vivo situation, and, vice versa, other cellular and molecular effects may be of greater importance in a therapeutic in vivo setting. More specifically, we and others delineate miR-506-mediated inhibition of cell cycle as a major contributor to tumor cell inhibition in vitro, especially in rapidly dividing cells with a fast cell cycle. However, while some cell-cycle-inhibitory and antiproliferative effects are also observed in vivo, the induction of apoptosis, autophagy, and necrosis plays a more important role in this context. This may be readily explained by the substantially lesser proliferation rate of tumor cells in the intact in vivo tissue environment as opposed to anchorage-dependent 2D cell culture of isolated tumor cells, and also highlights the necessity of studying effects in a relevant in vivo setting. Still, major effects and effectors identified in vitro are also found in vivo. As stated above, the higher relevance of inducing cell death/necrosis overproliferation inhibition in vivo may also well explain the seemingly later onset of therapeutic effects of miR506-3p replacement when only looking at tumor sizes and indicates that tumor size measurements rather underestimate actual therapeutic effects.

It should be noted that miR506-3p transfection/replacement mediates cell death through several different mechanisms, which are in part newly described in this study, including induction of apoptosis, necrosis, autophagy, and ROS. This is paralleled by alterations in expression levels of many different key proteins. These include the oncogene MYC [27], CDKs as mediators of cell cycle, as well as antiapoptotic proteins bcl-2 and bcl-xl. The miR-506 effect on MYC, mediated through the silencing of serine/threonine kinase 38 (STK38/NDR1) [27] and/or the effector of the Hippo pathway, the transcriptional coactivator YAP, may also explain our finding of survivin downregulation, since MYC has been described previously as connected to survivin transcription [28,29,30]. Beyond effects on expression levels, the downstream activation/inactivation of key players can be affected as well, as exemplified by bcl-2. The overexpression of IRS-1 has been shown to result in reduced phosphorylation of bcl-2 and subsequently enhanced anti-apoptotic activity of bcl-2. Vice versa, the downregulation of IRS 1, which was observed upon miR-506-3p in our study, can lead to activation of apoptotic pathways [31].

Downregulation of CDK4/CDK6 in combination with p21 upregulation are major regulators of cell cycle arrest and important for tumor growth reduction. The deregulation of cell-cycle-dependent proteins may thus contribute to therapeutic effects of miR506-3p in PDAC therapy as well. This is also supported by a recent study demonstrating miR-506 mediated downregulation of CDK1 and CDK4 [18]. Moreover, the induction of reactive oxygen species and autophagy, as well as of alterations in mitochondrial potential and structure, are additional steps towards cellular stress response and cell death. The production of reactive oxygen species has been correlated to miRNA expression signatures, with growing evidence of a miRNA/ROS reciprocal connection in cancer cells [32]. MiR506-3p was shown to negatively regulate the expression of NF-κB p65 with a subsequent increase in ROS levels, leading to p53 activation and increased apoptosis in lung cancer cells [33]. This pathway may thus contribute to ROS and apoptosis induction in PDAC as well.

Autophagy is a recycling process for nonessential cellular materials, using degradation processes to reduce cellular stress and nutrient deficiency [34], and has been identified as an important mechanism for PDAC growth [35]. However, its induction for longer time periods leads to autophagy-related cell death [17]. In our study, we demonstrate the induction of autophagy in PDAC upon replacement of miR506. Besides other mechanisms of action, survivin has also been shown to affect the conversion of the cytosolic LC3-I into the autophagic-vesicle-associated LC3-II form [36]. Reduced survivin levels may thus explain the altered LC3-II/LC3-I ratios found in our study in vitro and in vivo.

We also demonstrate that miR506-3p induces cellular senescence and alterations in mitochondrial potential and structure. In ovarian cancer cells, miR-506 has been shown to induce senescence by directly targeting the CDK4/6-FOXM1 axis. Moreover, the downregulation of SNAI2 by miR506-3p led to inhibition of EMT with altered morphology of the cells [6]. This EMT effect could explain the altered morphology of PaTu-8988t cells and the elevated granularity of the cells observed in our study. We also observed alterations in mitochondria morphology from a more fragmented state to an elongated tubular structure. Under normal conditions, mitochondria undergo continuous cycles of fusion and fission. Notably, the protein DYN2 (dynamin 2) is important for the last step of the fission process [37] and in silico analysis predicts DYN2 as a direct target of miR506-3p, which may well explain the inhibition of the fission process. Moreover, mitochondria morphology changes during cell cycle, from the elongated status in G1/S and the fragmented form in the early M phase [38]. This fragmentation is induced by the phosphorylation of RALA (Ras-like GTPase) by Aurora A, with subsequent complexation with RALBP1 and rearrangement to the mitochondria surface [39]. Interestingly, Aurora A, RALA, and RALBP1 are in silico predicted targets of the miRNA-506-3p as well. Inhibition of this fragmentation pathway has been shown to inhibit the mitochondrial fission process, and led to reduced cellular proliferation, impaired S-phase transition [40], and partial cellular G2-/M-phase arrest [41]. Finally, the elimination of mitochondria via mitophagy is linked to the fission and fusion event, and to hyperpolarization of the mitochondria as well. Since fission is required for mitophagy [42], fission blockade by miR-506-3p-mediated downregulation of important proteins can well explain the hyperpolarized status of the elongated mitochondria.

While our study clearly demonstrates the functional relevance and potential therapeutic usefulness of miR506-3p, it should still be noted that current data rely on cell lines and corresponding xenografts. Thus, results will have to be validated in models that are closer to the patient, e.g., patient-derived xenograft (PDX)-bearing mice or tissue slice cultures from primary tumors. In addition, while profound tumor-inhibitory effects are observed, miR506-3p replacement was found to be not strong enough for complete tumor regression. This indicates the potential usefulness of combination therapies, by combined application of miR506-3p with established cytostatics or targeted inhibitors, or in a sequential therapy regime. This will have to be explored in subsequent studies.

Taken together, our study highlights miRNA replacement as a powerful method by simultaneously influencing multiple cellular and molecular pathways, with modulation of oncogene and suppressor gene expression [43] and thus also addressing the issue of cancer as a pathway disease [44]. Due to its broad mechanisms of action on very relevant target genes, miR506-3p is identified as a particularly powerful tumor-inhibitory miRNA in PDAC. More recently, the clinical use of small RNAs as novel drug entities has become reality, with the first siRNA drugs being approved and several other small RNA therapeutics being investigated at the clinical stage (see [45] for review). The failure of the first tumor-targeted miRNA-based drug (the miR-34a mimic MRX34 [46]) also emphasizes the importance of identifying optimal miRNAs for replacement and suitable formulations for their delivery. While the latter aspect still must be considered as a major bottleneck as well, the nanoparticle field has shown progress in the development of systems for small RNA delivery (see [47,48] for review), and our system proved to be efficient in this regard.

## Data Availability

Data is contained within the article or supplementary material.

## Supplementary Materials

The following supporting information can be downloaded at: https://www.mdpi.com/article/10.3390/biomedicines10071692/s1, Figure S1: (A) Expression levels of miRNA-506-3p, in various pancreatic carcinoma cell lines. (B) Relative mRNA levels of ROS-related genes GOT1, Nrf2, Ck2a, and PTGR2 after 72 h of miR506-3p vs. NC1 transfection in PaTu-8988t cells. (C) Relative mRNA levels of cell-cycle-related cyclins CDK1, CDK2, CDK4, and CDK6 after 120 h of miR506-3p vs. NC1 transfection in Colo357 cells. Figure S2: Original data from the cell lines Colo357 and Panc89 with apoptosis (A), necrosis (N), and debris (D) indicated. Figure S3: Analysis of autophagic flux by determining p62 protein levels. Left: original Western blot data; right: quantitation of p62 protein levels normalized to GAPDH. Table S1: Sequences of primers used in this study; Table S2: Western blot antibodies and dilutions used in this study; Table S3: Primary antibodies for immunohistochemistry used in this study.
