# Supplementary material for: Therapeutic miR-506-3p Replacement in Pancreatic Carcinoma Leads to Multiple Effects including Autophagy, Apoptosis, Senescence, and Mitochondrial Alterations In Vitro and In Vivo
Source: Biomedicines. 2022 Jul 13;10(7):1692. doi: 10.3390/biomedicines10071692 (PMC9312874; doi:10.3390/biomedicines10071692)
Supplement: Supplementary file 1 [file biomedicines-10-01692-s001.zip › Borchardt et al - Biomedicine revised - Figure S1.pptx]

## Slide 1
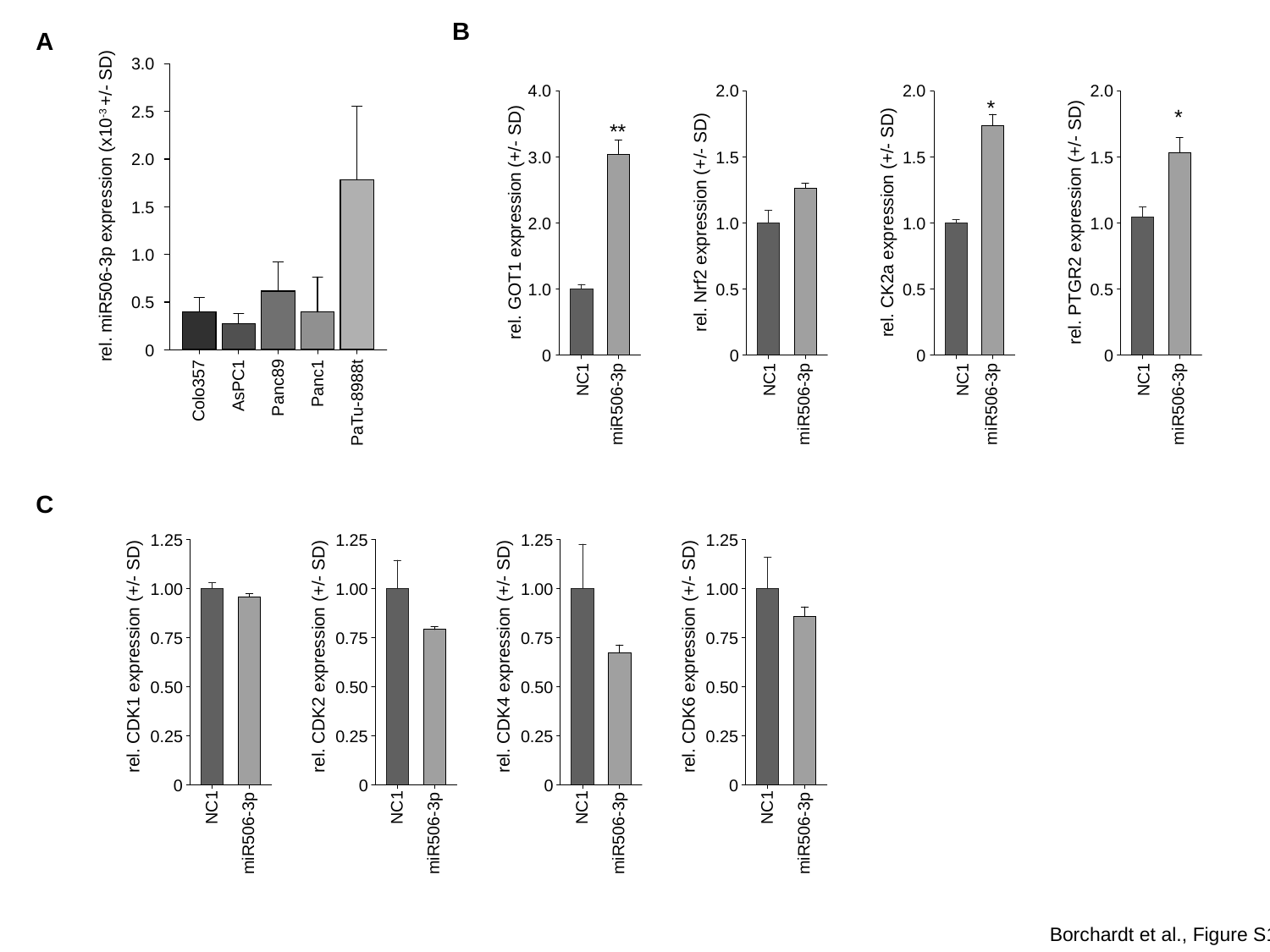

B
A
3.0
4.0
**
3.0
2.0
1.0
0
NC1
miR506-3p
rel. GOT1 expression (+/- SD)
2.0
1.5
rel. Nrf2 expression (+/- SD)
1.0
0.5
0
NC1
miR506-3p
2.0
*
1.5
rel. CK2a expression (+/- SD)
1.0
0.5
0
NC1
miR506-3p
2.0
*
1.5
rel. PTGR2 expression (+/- SD)
1.0
0.5
0
NC1
miR506-3p
2.5
2.0
rel. miR506-3p expression (x10-3 +/- SD)
1.5
1.0
0.5
0
Panc1
AsPC1
Panc89
Colo357
PaTu-8988t
C
1.25
1.25
1.25
1.25
1.00
1.00
1.00
1.00
0.75
0.75
0.75
0.75
rel. CDK1 expression (+/- SD)
rel. CDK2 expression (+/- SD)
rel. CDK4 expression (+/- SD)
rel. CDK6 expression (+/- SD)
0.50
0.50
0.50
0.50
0.25
0.25
0.25
0.25
0
0
0
0
NC1
NC1
NC1
NC1
miR506-3p
miR506-3p
miR506-3p
miR506-3p
Borchardt et al., Figure S1
