# Supplementary material for: Therapeutic miR-506-3p Replacement in Pancreatic Carcinoma Leads to Multiple Effects including Autophagy, Apoptosis, Senescence, and Mitochondrial Alterations In Vitro and In Vivo
Source: Biomedicines. 2022 Jul 13;10(7):1692. doi: 10.3390/biomedicines10071692 (PMC9312874; doi:10.3390/biomedicines10071692)
Supplement: Supplementary file 1 [file biomedicines-10-01692-s001.zip › Borchardt et al - Biomedicine revised - Figure S3.pptx]

## Slide 1
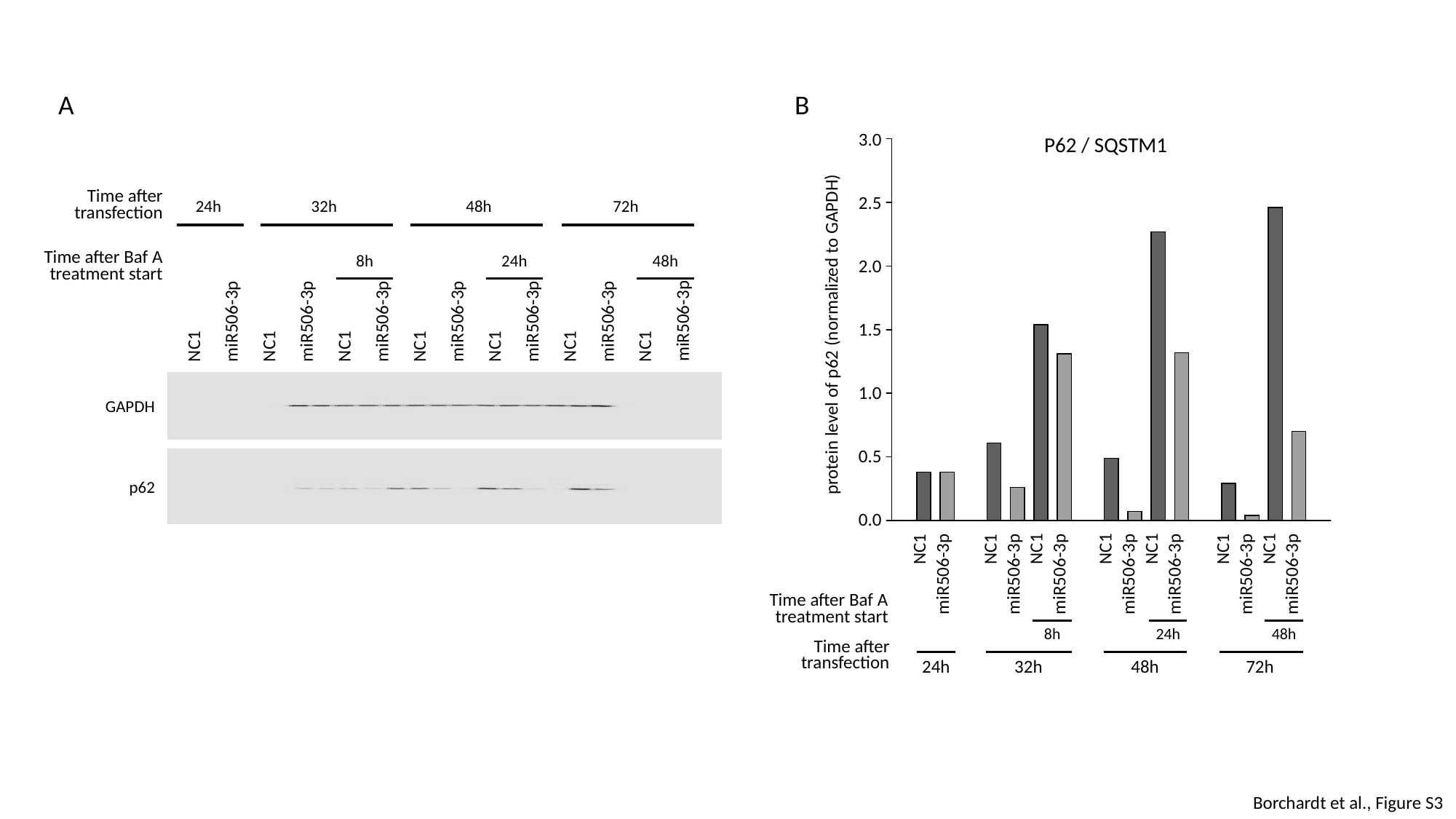

A
B
3.0
P62 / SQSTM1
protein level of p62 (normalized to GAPDH)
Time after transfection
2.5
24h
32h
48h
72h
Time after Baf A treatment start
8h
24h
48h
2.0
miR506-3p
miR506-3p
miR506-3p
miR506-3p
miR506-3p
miR506-3p
miR506-3p
1.5
NC1
NC1
NC1
NC1
NC1
NC1
NC1
1.0
GAPDH
0.5
p62
0.0
NC1
miR506-3p
NC1
miR506-3p
NC1
miR506-3p
NC1
miR506-3p
NC1
miR506-3p
NC1
miR506-3p
NC1
miR506-3p
Time after Baf A treatment start
8h
24h
48h
Time after transfection
24h
32h
48h
72h
Borchardt et al., Figure S3
