# Supplementary material for: Therapeutic miR-506-3p Replacement in Pancreatic Carcinoma Leads to Multiple Effects including Autophagy, Apoptosis, Senescence, and Mitochondrial Alterations In Vitro and In Vivo
Source: Biomedicines. 2022 Jul 13;10(7):1692. doi: 10.3390/biomedicines10071692 (PMC9312874; doi:10.3390/biomedicines10071692)
Supplement: Supplementary file 1 [file biomedicines-10-01692-s001.zip › Borchardt et al - Biomedicine revised - Table S1.pdf]

Table S1. Sequences of primers used in this study

Gene	Forward-primer	Reverse-primer
**CDK1**	CATGGGGATTCAGAAATTGA	ATTCGTTTGGCTGGATCATA
**CDK2**	AAGCCAGAAACAAGTTGACG	GAAGAGGAATGCCAGTGAGA
**CDK4**	GAGTGTGAGAGTCCCCAATG	ATGCTCAAACACCAGGGTTA
**CDK6**	CAGGGGATTTTCATGTTGAG	GCACATCAACGGAATTTTTC
**CK2a**	TCATGAGCACAGAAAGCTACGA	AATGGCTCCTTCCGAAAGATC
**GOT1**	GTCCAGTACCACCAAAGTAGTTCTC	GGCTCTAATCCCAGTCTCCAAA
**miRNA-506-3p**	CGTAAGGCACCCTTCTGA	Universal PCR Primer (Quanta)
**Nrf2**	GCATGATGCCCAATGTGAGA	TCCAAGCGGCTTGAATGTTT
**PTGR2**	TTGGCAGCTATCTCAAGTCG	TGTGTTTGCTTTCTTCTATAATTCCAATAC
**SNORD44**	GCAAATGCTGACTGAACATGAA	Universal PCR Primer (Quanta)
**β-actin**	CCAACCGCGAGAAGATGA	CCAGAGGCGTACAGGGATAG
