# Supplementary material for: Therapeutic miR-506-3p Replacement in Pancreatic Carcinoma Leads to Multiple Effects including Autophagy, Apoptosis, Senescence, and Mitochondrial Alterations In Vitro and In Vivo
Source: Biomedicines. 2022 Jul 13;10(7):1692. doi: 10.3390/biomedicines10071692 (PMC9312874; doi:10.3390/biomedicines10071692)
Supplement: Supplementary file 1 [file biomedicines-10-01692-s001.zip › Borchardt et al - Biomedicine revised - Table S2.pdf]

Table S2. Western blot antibodies and dilutions used in this study

Target protein	Dilution in 5% milk (TBST)	Cat number (vendor)
**Active Caspase3**	1:1000	#JM-3015-100 (MBL)
**β-actin**	1:5000	#4970 (Cell Signaling)
**Bcl2**	1:2000	#15071 (Cell Signaling)
**Bcl-xL**	1:1000	#2764 (Cell Signaling)
**CDK6**	1:1000	#3136 (Cell Signaling)
**GAPDH**	1:5000	ab181602 (Abcam)
**IRS1**	1:1000	#2382 (Cell Signaling)
**LC3B**	1:1000	#3868 (Cell Signaling)
**P62**	1:1000	#88588 (Cell Signaling)
**P53**	1:2000	#9282 (Cell Signaling)
**P21**	1:1000	#2947 (Cell Signaling)
**PCNA**	1:1000	#13110 (Cell Signaling)
**PLK1**	1:1000	#4513 (Cell Signaling)
**Survivin**	1:5000	ab76424 (Abcam)
