# Supplementary material for: Therapeutic miR-506-3p Replacement in Pancreatic Carcinoma Leads to Multiple Effects including Autophagy, Apoptosis, Senescence, and Mitochondrial Alterations In Vitro and In Vivo
Source: Biomedicines. 2022 Jul 13;10(7):1692. doi: 10.3390/biomedicines10071692 (PMC9312874; doi:10.3390/biomedicines10071692)
Supplement: Supplementary file 1 [file biomedicines-10-01692-s001.zip › Borchardt et al - Biomedicine revised - Table S3.pdf]

Table S3. Primary antibodies for immunohistochemistry used in this study

Target protein	Dilution	Buffer
**Active Caspase3 (#JM-3015-100)**	1:100	Citrate-Buffer pH 6.0
**Ki-67 (M7240, Dako, MIB-1)**	1:100	EDTA-Buffer pH 8.0
**Survivin (ab76424)**	1:500	Tris-EDTA Buffer pH 9.0
**c-myc**	1:200	EDTA-Buffer pH 8.0
**LC3B**	1:100	EDTA-Buffer pH 8.0
