# Supplementary figures and images for: Therapeutic miR-506-3p Replacement in Pancreatic Carcinoma Leads to Multiple Effects including Autophagy, Apoptosis, Senescence, and Mitochondrial Alterations In Vitro and In Vivo
Source: Biomedicines. 2022 Jul 13;10(7):1692. doi: 10.3390/biomedicines10071692 (PMC9312874; doi:10.3390/biomedicines10071692)

## Slide 1
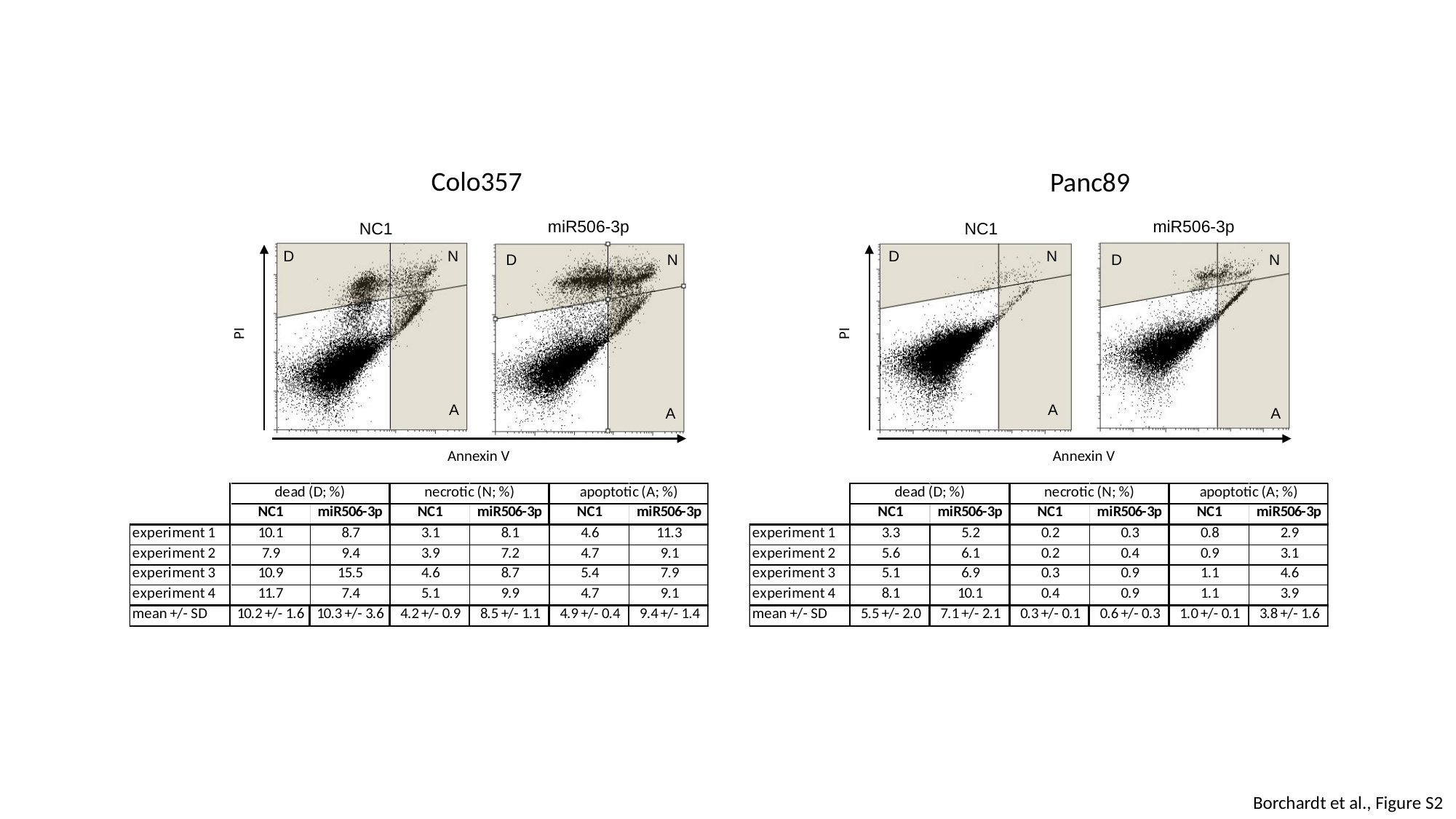

Colo357
miR506-3p
NC1
D
N
D
N
PI
A
A
Annexin V
Panc89
miR506-3p
NC1
D
N
D
N
PI
A
A
Annexin V
Borchardt et al., Figure S2

Supplement: Supplementary file 1 [file biomedicines-10-01692-s001.zip › Borchardt et al - Biomedicine revised - Figure S2.pptx]
